# Evaluation of a novel consultant psychiatric clinic in general practices and its effects on secondary mental health contact and the general practitioners’ perspectives

**DOI:** 10.1192/bjo.2021.202

**Published:** 2021-06-18

**Authors:** Daniel Whitney, Daniel Whitney, Guy Brookes

**Affiliations:** 1 ST6 in General Adult Psychiatry, Tees, Esk and Wear Valleys NHS Foundation Trust; 2 Tees Esk and Wear Valleys NHS Foundation Trust; 3 Leeds and York Partnership Foundation Trust

## Abstract

**Aims:**

To assess whether direct access to a 45 minute screen appointment in a Consultant Psychiatric clinic, based in General Practice, affects; the number of contacts patients have with secondary care pre and post being seen; whether the General Practitioner (GP) would have referred to secondary services if the clinic had not been in operation; the GPs’ views on how helpful the clinic was in understanding the patients’ problems and managing the problems outside of secondary care.

**Background:**

A Consultant Psychiatrist in Leeds offered bespoke 45 minute screening appointment clinics in three sister GP practices, accepting direct referrals from GPs without requiring referrals to the local Community Mental Health Team (CMHT). This model was created to reduce the number of patients moving repeatedly between GP and secondary mental health services as this was leading to patient dissatisfaction and increased GP and CMHT workloads.

**Method:**

We compared the number of mental health contacts (per month), for each of the 57 patients who had been referred to the clinic, in the months pre and post being seen in the clinic. We also asked the involved GPs to complete a brief survey for each patient who had been referred to determine whether, they would otherwise have been referred to the CMHT and whether the clinic has helped with their understanding and management of the patients’ problems.

**Result:**

The mean number of contacts with secondary services before being seen in clinic was 3.30 per month compared to 0.44 after being seen. The mean difference of 2.86 is statistically significant on a paired-test with a P Value of 0.0149 (95% confidence intervals of 0.58 to 5.13). We received 22 survey responses from GPs of patients referred to the clinic including for patients who did not attend. All 22 responses indicated that the patient would have been referred to the CMHT if the clinic had not been available. 95% were rated as being very helpful or moderately helpful in understanding the patient's problems. 91% were rated as very helpful or moderately helpful in managing the patients’ problems outside secondary care.

**Conclusion:**

Our evaluation has demonstrated that a model of direct access for GPs to a Consultant Psychiatric clinic can reduce referrals and patient contacts with secondary mental health services. GPs have found this model helpful in understanding patients’ problems and managing the problems outside of secondary care.

